# Supplementary material for: Causal Relationship between Aging and Anorexia Nervosa: A White-Matter-Microstructure-Mediated Mendelian Randomization Analysis
Source: Biomedicines. 2024 Aug 16;12(8):1874. doi: 10.3390/biomedicines12081874 (PMC11351342; doi:10.3390/biomedicines12081874)
Supplement: Supplementary file 1 [file biomedicines-12-01874-s001.zip › Supplementary Figure/Figure S4/Singe SNP plot of MR analysis for TL to ubm-b-1744.pdf]

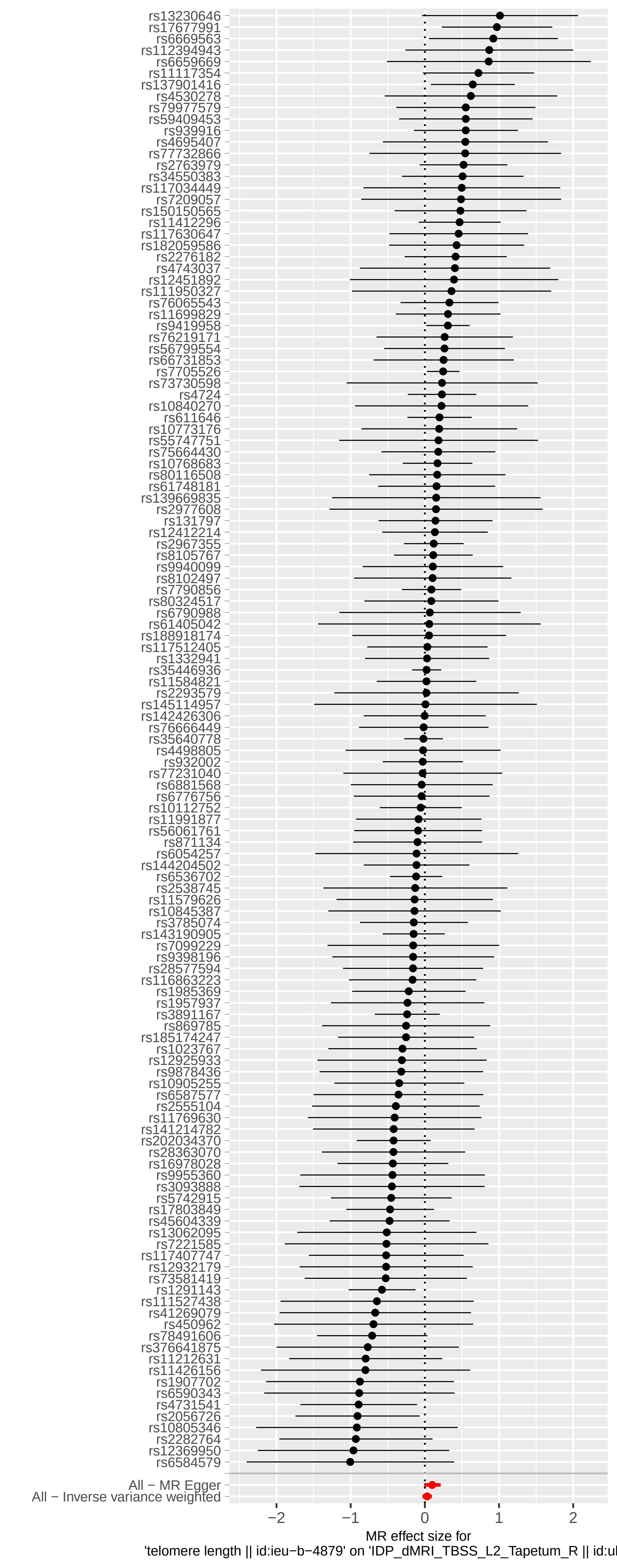
rs13230646
rs17677991
rs6669563
rs112394943
rs6659669
rs11117354
rs137901416
rs4530278
rs79977579
rs59409453
rs939916
rs4695407
rs77732866
rs2763979
rs34550383
rs117034449
rs7209057
rs150150565
rs11412296
rs117630647
rs182059586
rs2276182
rs4743037
rs12451892
rs111950327
rs76065543
rs11699829
rs9419958
rs76219171
rs56799554
rs66731853
rs7705526
rs73730598
rs4724
rs10840270
rs611646
rs10773176
rs55747751
rs75664430
rs10768683
rs80116508
rs61748181
rs139669835
rs2977608
rs131797
rs12412214
rs2967355
rs8105767
rs9940099
rs8102497
rs7790856
rs80324517
rs6790988
rs61405042
rs188918174
rs117512405
rs1332941
rs35446936
rs11584821
rs2293579
rs145114957
rs142426306
rs76666449
rs35640778
rs4498805
rs932002
rs77231040
rs6881568
rs6776756
rs10112752
rs11991877
rs56061761
rs871134
rs6054257
rs144204502
rs6536702
rs2538745
rs11579626
rs10845387
rs3785074
rs143190905
rs7099229
rs9398196
rs28577594
rs116863223
rs1985369
rs1957937
rs3891167
rs869785
rs185174247
rs1023767
rs12925933
rs9878436
rs10905255
rs6587577
rs2555104
rs11769630
rs141214782
rs202034370
rs28363070
rs16978028
rs9955360
rs3093888
rs5742915
rs17803849
rs45604339
rs13062095
rs7221585
rs117407747
rs12932179
rs73581419
rs1291143
rs111527438
rs41269079
rs450962
rs78491606
rs376641875
rs11212631
rs11426156
rs1907702
rs6590343
rs4731541
rs2056726
rs10805346
rs2282764
rs12369950
rs6584579
All − MR Egger
All − Inverse variance weighted
−2
−1
0
1
2
MR effect size for
'telomere length || id:ieu−b−4879' on 'IDP_dMRI_TBSS_L2_Tapetum_R || id:ul
